# Corrected Genome Sequence of *Acinetobacter baumannii* Strain AB0057, an Antibiotic-Resistant Isolate from Lineage 1 of Global Clone 1

**DOI:** 10.1128/genomeA.00836-17

**Published:** 2017-08-31

**Authors:** Mohammad Hamidian, Pratap Venepally, Ruth M. Hall, Mark D. Adams

**Affiliations:** a School of Life and Environmental Sciences, The University of Sydney, Sydney, New South Wales, Australia; b J. Craig Venter Institute, Rockville, Maryland, USA; c J. Craig Venter Institute, La Jolla, California, USA

## Abstract

Extensively antibiotic-resistant *Acinetobacter baumannii* isolate AB0057 recovered in the United States in 2004 was one of the first global clone 1 isolates to be completely sequenced. Here, the complete 4.05-Mb genome sequence (chromosome and one plasmid) has been revised using Illumina HiSeq data and targeted sequencing of PCR products.

## GENOME ANNOUNCEMENT

The extensively antibiotic-resistant *Acinetobacter baumannii* isolate AB0057 was recovered in 2004 from the bloodstream of a patient at Walter Reed Army Medical Center (1, 2). It is susceptible to only colistin (3) and the aminoglycosides tobramycin and amikacin (1), as well as netilmicin and rifampin. AB0057 was the second global clone 1 (GC1) strain to be completely sequenced (1). AB0057 belongs to CC1 (sequence type 1 [ST1]) in the Institute Pasteur MLST Scheme (4) and to ST207 in the Oxford MLST Scheme (5) and carries the KL4 capsule genes and OCL3 at the outer core locus (6). Most of the resistance genes, *tetA*(A), *catA1*, *bla*_TEM_, *aphA1b*, *aacC1*, *aadA1*, and *sul1*, are located in the AbaR3 resistance island in the *comM* gene (1). The carbapenemase-encoding *oxa23* gene is in AbaR4 (1). Tn*6168* carrying a second copy of *ampC* confers resistance to third-generation cephalosporins (7).

The previously published genome sequence was determined using a mix of 454 pyrosequencing and Sanger sequencing (1). Here, we report an improved sequence using Illumina sequence data. Libraries were prepared from whole-cell DNA and sequenced on an Illumina HiSeq system at the Australian Genome Research Facility. In total, 2.4 million 100-bp paired-end reads were obtained for an average read depth of ∼100×. Reads were assembled *de novo* using SPAdes version 3.4.0 with default parameters (8), and contigs were mapped onto the original assemblies (the first versions of GenBank accession no. CP001182 and CP001183). Protein-coding genes, rRNAs, and tRNAs were annotated using the NCBI Prokaryotic Genome Annotation Pipeline (https://www.ncbi.nlm.nih.gov/genome/annotation_prok), and the antibiotic resistance and polysaccharide biosynthesis loci, transposons, insertion sequences, and pAB0057 were annotated manually.

The revised contiguous chromosomal sequence comprised 4,055,148 bp (the second version of GenBank accession no. CP001182), compared with 4,050,513 bp in the original version. The final assembly incorporated published corrections to the intrinsic *ampC* gene (7). PCR followed by sequencing identified an additional copy of Tn*2006* carrying the *oxa23* carbapenem resistance gene in the chromosome (bases 2478007 to 2482811). In addition to the insertion sequence described previously (9), two copies of ISAba13 and a single copy of ISAba26 were also present. Two single base pairs missing from pAB0057, the only plasmid in AB0057, were added to the revised sequence (second version) of GenBank accession no. CP001183, now totaling 8,731 bp.

The original annotation included 3,790 protein-coding genes (CDS features). The revised annotation contains 3,777 CDS features. Seventy-four CDSs were removed, and 105 were added; 3,110 CDSs are identical between the two annotations, and 49 have the same length but differ at the nucleotide level. Corrections of small insertion/deletion errors resulted in a change of reading frame and/or merging and splitting of CDS regions such that 384 CDSs in the revised genome are longer and 131 are shorter than the corresponding CDSs in the original annotation.

This revised genome sequence of AB0057 will underpin studies of the genetics and evolution of the GC1 clonal complex.

### Accession number(s).

This complete genome sequence has been deposited in DDBJ/ENA/GenBank under the revised accession numbers of the AB0057 genome, CP001182 (chromosome) and CP001183 (pAB0057). The versions described in this paper are the second versions.
